# WHAT DO LONG-TERM CARE LEADERS SAY ABOUT THEIR PREPAREDNESS FOR COVID-19? A QUALITATIVE STUDY

**DOI:** 10.1093/geroni/igac059.1785

**Published:** 2022-12-20

**Authors:** Darren Liu, Sandi Lane, Betty Burston, Robert Rados

**Affiliations:** West Virginia University, Morgantown, West Virginia, United States; Appalachian State University, Boone, North Carolina, United States; University of Nevada Las Vegas, Nevada, Las Vegas, Nevada, United States; Southern Illinois University Carbondale, Carbondale, Illinois, United States

## Abstract

The post-acute and long-term care (PALTC) sector has, in some respects, served as the epicenter in the U.S. during the COVID-19 pandemic. Many decisions were carried out by administrative and/or clinical leaders during this pandemic. The decisions were made based on their professional experiences, and recommendations by the Centers for Medicare & Medicaid Services (CMS). However, little research has reported on the perspective of those administrators who took the lead during this most difficult time. This study aims to understand how responses and decisions were formed during COVID-19 to ensure resources were available protect staff and residents. Accordingly, this study tried to answer two key questions: 1) What did the PALTC administrative and clinical leaderships learn? 2) What can we do better not if, but when COVID ever “…hits again?” We interviewed nursing home and/or assisted living administrators in two conveniently selected states: Pennsylvania and North Carolina. These interviews (each of which took about 30 minutes) were conducted over Zoom using structured and open-ended questions. The transcripts were entered and analyzed using NVivo – a qualitative data analysis software. The results revealed several themes including communications, relationship building, experience as an administrator, fears and resilience, as well as successful activities to support their staffs such as recognition, bonuses, and food bags prepared for their family. The findings highlight some important administrators’ thoughts which recommend key future strategies. These include whether preparedness assets, knowledge, resources, and policies were adequate and where the future efforts should focus.

